# Mitochondrial Dysfunction in CD4+ T Effector Memory RA+ Cells

**DOI:** 10.3390/biology12040597

**Published:** 2023-04-14

**Authors:** Marie Strickland, Salanne Lee, Shi Yong Neo, Akhila Balachander, Ivy Low, Seri Mustafah, Wah Ing Goh, Graham D. Wright, Anis Larbi, Sylvia L. F. Pender

**Affiliations:** 1 Singapore Immunology Network (SIgN), Agency for Science, Technology and Research (A*STAR), 8A Biomedical Grove, Immunos, Singapore 138648, Singapore; 2 School of Clinical and Experimental Sciences, Faculty of Medicine, University of Southampton, Southampton SO17 1BJ, UK; 3 Research Support Centre (RSC), Agency for Science, Technology and Research (A*STAR), 30 Biopolis Street, Matrix Building, Singapore 138671, Singapore

**Keywords:** metabolism_1_, T cells, terminally differentiated effector memory T cells (TEMRA), stimulation, flow cytometry

## Abstract

**Simple Summary:**

The mitochondria is the powerhouse of the cell. This powerhouse becomes somehow dysfunctional as we get older; hence, our immune responses to infection become poor, resulting in poor vaccine efficiency. This factor is essential when designing vaccines for the elderly. In this study, we aim to study the biology of specific immune cells and understand why and how they exhibit different mitochondrial phenomena and dynamics when confronted with stimulation following infection.

**Abstract:**

Human ageing is accompanied by poor responses to infection and decreased vaccine efficacy. While the causes of this can be attributed to defects in the immune system that increase with age, it is unknown whether mitochondrial dysfunction may also contribute to these phenomena. This study aims to assess mitochondrial dysfunction in CD4+ terminal effector memory T cells re-expressing CD45RA (TEMRA) cells and other CD4+ memory T cell subtypes, which are increased in number in the elderly population, with respect to how their metabolic responses to stimulation are altered compared to CD4+ naïve T cells. In this study, we show that CD4+ TEMRA cells exhibit altered mitochondrial dynamics compared to CD4+ naïve cells and CD4+ central and effector memory cells, with a 25% reduction in OPA1 expression. CD4+ TEMRA and memory cells show increased upregulation of Glucose transporter 1 following stimulation and higher levels of mitochondrial mass compared to CD4+ naïve T cells. Additionally, TEMRA cells exhibit a decrease in mitochondrial membrane potential compared to other CD4+ memory cell subsets by up to 50%. By comparing young to aged individuals, more significant mitochondria mass and lower membrane potential were observed in CD4+ TEMRA of young individuals. In conclusion, we suggest that CD4+ TEMRA cells may be impaired with respect to their metabolic response to stimulation, possibly contributing to impaired responses to infection and vaccination.

## 1. Introduction

Poor vaccine efficacy and poor responses to infection are two major health concerns affecting the elderly population. These concerns have both been linked to the ageing of the CD4+ T cell compartment [1]. The ability of T cells to adapt their metabolism to fulfil their effector activity is integral to T cell function [2]. Before stimulation, T cells exist in a quiescent state with limited metabolic requirements in order to maintain migration and homeostatic proliferation. Following stimulation, T cells must proliferate rapidly and transform into effector cells. This increased demand for cell growth, division and differentiation requires both ATP and biosynthesis derived from increased metabolism. Both CD4+ and CD8+ T cells prioritise oxidative phosphorylation and fatty acid oxidation in the quiescent state to generate ATP and support homeostatic proliferation, rapidly switching to aerobic glycolysis following stimulation [3]. In the elderly population, it is unknown how this process is affected, and if it may contribute to the poor T cell responses observed in this population.

The CD4+ and CD8+ T cell populations can be subdivided based on their expression of CD27 and CD45RA, revealing four populations. Naïve T cells express high levels of CD27 and CD45RA, while acquiring memory leads to loss of CD45RA in central memory (CM) T cells and further co-loss of CD27 expression in effector memory (EM) T cells. Furthermore, the CD27- population can be divided into two populations: those which lack expression of CD45RA represent the EM subset and those which regain CD45RA expression are termed terminal EM T cells re-expressing CD45RA (TEMRA). These populations are analogous to those identified based on CCR7 and CD45RA expression [4,5]. Roughly 60% of TEMRA cells express high levels of senescent markers, such as killer-cell lectin-like receptor G1 (KLRG1), CD57 and programmed cell death receptor 1 (PD1), compared to only 10% of naïve and CM cells [6,7]. On the other hand, TEMRA cells, especially those which are CD8+, express high levels of cytotoxic cytokines TNF-α, IFN-γ, perforin and granzyme B, showing that these cells have a distinct phenotype compared to EM T cells [4,7]. These TEMRA cells possess a lower proliferative capacity and reduced telomerase activity compared to their EM counterparts [8].

TEMRA cells have previously been shown to represent a senescent population of cells that accumulate with age and as a result of specific infections such as cytomegalovirus [6,9]. Approximately 22% of the CD8+ T cell population consists of TEMRA cells, while within the CD4+ T cell population, this subset is much rarer, contributing about 5% of total CD4+ T cells [8]. T cell compartmentalisation changes dramatically with age, with an increasing CD4: CD8 ratio and an increase in the number of memory T cells in the periphery [10]. For example, the number of CD8+ TEMRA cells increases from approximately 30% in individuals less than 40 years old to 60% in individuals over 90 years old, whereas CD8+ naïve cells decrease from 40% to 5% in these individuals [6]. It is worth noting that changes in the CD4+ T cell population are often not as marked as they are for the CD8+ T cell population [6]. CD8+ and CD4+ TEMRA develop a senescence-associated secretory phenotype (SASP) characterised by the secretion of pro-inflammatory cytokines, chemokines and proteases. The links between SASP and metabolic signalling have been explored previously, with low levels of inflammation driven by SASP contributing to various age-related disorders [11,12].

Mitochondrial dysfunction, a key hallmark in many age-related diseases, has previously been observed in CD8+ TEMRA [4], and is characterised by reduced mitochondrial mass compared to EM T cells and reduced mitochondrial membrane potential. Additionally, CD8+ TEMRA have been shown to have similar levels of mitochondrial mass compared to CD8+ naïve T cells [13]. As increased phosphorylation of AMP-activated protein kinase (AMPK) has been observed in CD4+ TEMRA but not CD8+ TEMRA, it has been suggested that these cells may behave differently [8,14]. Increased AMPK phosphorylation often results in higher mitochondrial mass in CD4+ TEMRA cells than in CD8+ TEMRA, indicating that CD8+ TEMRA cells may be prone to mitochondrial dysfunction [15]. While studies on CD4+ TEMRA are limited, it has been noted that CD4+ TEMRA cells have double the mitochondrial mass of CD8+ TEMRA cells, in addition to increased spare respiratory capacity, slowing their rate of senescence [16].

Mitochondrial organisation is governed by the expression of mitochondrial fusion proteins, such as mitofusin 1 and mitofusin 2 (MFN1 and MFN2), and mitochondrial fission proteins, such as dynamin-related protein 1 (DRP1) and optic atrophy protein 1 (OPA1). These proteins act in synergy to organise the mitochondria in response to metabolic needs, promoting mitochondrial fusion and the growth of mitochondria when bioenergetic demands are high and promoting mitochondrial fission and the budding of mitochondria when metabolic demands are low [17,18,19]. While DRP1, MFN1 and MFN2 all regulate the outer mitochondrial membrane, OPA1 is responsible for the structure and organisation of the inner mitochondrial membrane known as the cristae and in turn the proximity of the components of the electron transport chain [19]. Alterations in the levels of these proteins and mitochondrial disorganisation have long been associated with ageing and age-related diseases and increased inflammation [20,21].

CD4+ TEMRA cells are relatively unknown compared to CD8+ TEMRA cells and their naïve and CM/EM counterparts. As the CD4+ TEMRA cell compartment increases with age, there may be some link between TEMRA cells and the lack of response to infection and vaccination in the elderly population. Therefore, in this study, we aim to examine the metabolic and mitochondrial landscape in CD4+ TEMRA cells in order to delineate this question. In this study, we investigate the dynamics of mitochondrial fission and fusion, the intermembrane space of mitochondria, and how this affects mitochondrial dynamics and function in CD4+ T cell subsets. Additionally, a comparative analysis of peripheral blood from young versus elderly individuals revealed differential mitochondria mass and membrane potential in both CD4 and CD8 T cell subsets.

## 2. Materials and Methods

### 2.1. Subjects and Ethics Statement

Peripheral blood mononuclear cells (PBMCs) were collected from anonymised healthy adult volunteers and provided by the Blood Donation Centre, Health Sciences Authority in Singapore. Aged healthy individuals were defined as being above the age of 60 years old without the presence of co-morbidities. All subjects provided written informed consent, and the study was approved by the National University of Singapore–Institution Review Board (NUS-IRB 04-140).

### 2.2. Blood Samples

Purified mononuclear cells were isolated from whole blood using a Ficoll gradient for density separation and centrifuged at 400× *g* for 20 min at room temperature. Following isolation, cells were used fresh. For cell sorting, cells were frozen in FBS containing 10% DMSO for long-term storage and defrosted before cell sorting.

### 2.3. Antibodies and Reagents

Primary mouse monoclonal antibodies against CD3 (Clone: UHCT1), CD4 (Clone: OKT4), CD8 (Clone: SK1), CD27 (Clone: O323), CD28 (Clone: CD28.2), CD45RO (Clone: UCHL1) and CD45RA (Clone: HI100) were purchased from BioLegend (San Diego, CA, USA). Primary antibodies against GLUT1 (Host: Rabbit), OPA1 (Host: Mouse IgG1), MFN2 (Host: Mouse IgG2β) and DRP1 (Host: Mouse IgG2β) were purchased from Abcam (Cambridge, UK). Secondary antibodies against Mouse IgG1 (Host: Goat) and IgG2β (Host: Rat) were purchased from Abcam and R&D Systems, respectively. Mitochondrial dyes MitoTracker Green and TMRM were purchased from Thermo Fisher Scientific and Sigma-Aldrich, respectively.

### 2.4. Cell Culture and Flow Cytometry

**Figure 1:**
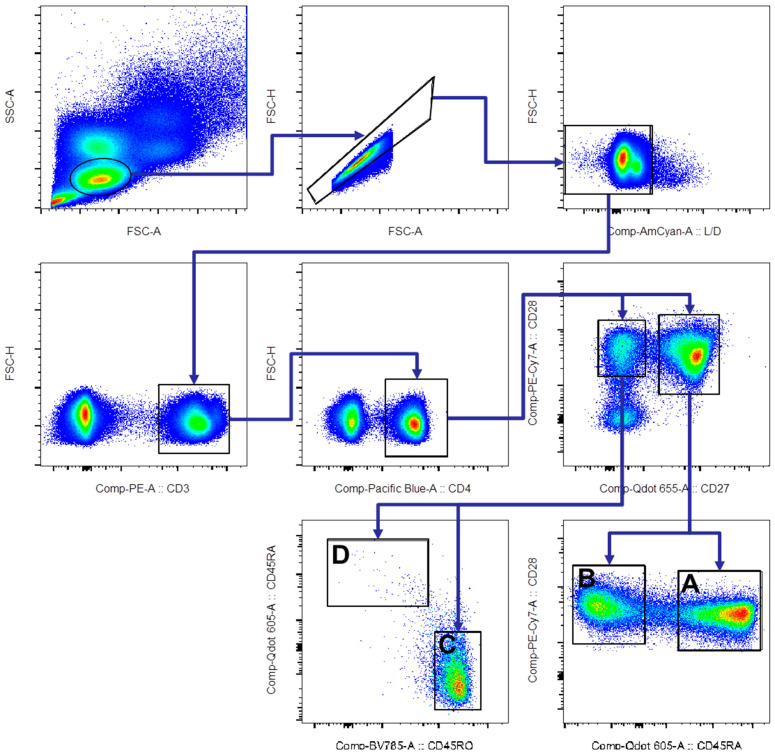
Gating and sorting strategy for CD4+ T cells gated for FSC/SSC, singlets, live cells, CD3+ and CD4+. Subsets gated as (**A**) naïve: CD28+ CD27+ and CD45RA+; (**B**) central memory: CD28+ CD27+ CD45RA-; (**C**) effector memory: CD28+ CD27- CD45RO+ CD45RA-; and (**D**) TEMRA: CD28+ CD27- CD45RO- CD45RA+ (*n* = 1; representative donor).

PBMCs were plated in a 96-well flat-bottom plate and cultured in RPMI-1640 supplemented with 10% FBS at a density of 2 × 10^6^ cells per well. Cells were stimulated with 96-well plates coated with anti-CD3 (Clone: OKT3; 2 µg/mL, Biolegend, San Diego, CA, USA) or PBS control and measured at 4 h and 24 h timepoints at 37 °C/5% CO_2_. After stimulation, cells were stained with cell surface markers anti-CD3, -CD4, -CD27, -CD28, -CD45RO and -CD45RA, permeabilized and then stained with anti-GLUT1, anti-OPA1, anti-MFN2 and anti-DRP1 (Abcam, Cambridge, UK). Cell viability was assessed using Live/Dead Aqua (ThermoFisher, Waltham, MA, USA). Cells were analysed using the BD LSRFortessa^TM^ X-20 cell analyser (BD Biosciences, Franklin Lakes, NJ, USA). Single-stained BD CompBeads were used to define compensation parameters. T-cell subsets were identified using the gating strategy observed in Figure 1.

### 2.5. Quantification of Mitochondrial Mass and Membrane Potential

PBMCs were plated in a 96-well flat-bottom plate and cultured in RPMI-1640 supplemented with 10% FBS at a density of 2 × 10^6^ cells per well. Cells were stimulated with coated anti-CD3 (Clone: OKT3; 2 µg/mL, Biolegend, San Diego, CA, USA) or PBS control and measured at 4 h and 24 h time points. For the final one hour of stimulation MitoTracker Green (100 nm, Thermo Fisher Scientific, Waltham, MA, USA) and TMRM (25 nM, Sigma-Aldrich, St. Louis, MI, USA) were added to the wells at 37 °C/5% CO_2_. After culture, cells were stained with cell surface markers anti-CD3, -CD4, -CD27, -CD28, -CD45RO and -CD45RA (BioLegend, San Diego, CA, USA). Cell viability was assessed using Live/Dead Aqua (ThermoFisher, Waltham, MA, USA). Cells were immediately analysed using the BD LSRFortessa^TM^ X-20 cell analyser (BD Biosciences, Franklin Lakes, NJ, USA). Separately stained cells were used to define compensation parameters.

### 2.6. Cell Sorting and Super-Resolution Microscopy of Mitochondria

CD3+ cells were purified from the PBMC pool using anti-CD3 coated beads and MACS technology before cell sorting (Miltenyi Biotech, Bergisch Gladbach, Germany). Following magnetic separation, cells were collected in a 5 mL polypropylene tube for staining. Cells were stained with anti-CD4, -CD8, -CD27, -CD28, -CD45RO and -CD45RA (BioLegend, San Diego, CA, USA) for 20 min. Cells were immediately sorted using the BD FACSAria II system (BD Biosciences). Separately stained BD CompBeads were used to define compensation parameters. After sorting, cells were plated into 96-well U-bottom plates at a density of 5 × 10^5^ per well and incubated at 37 °C/5% CO_2_ for 12 h.

Cells were seeded on #1.5H glass coverslips, fixed and permeabilised for staining using anti-TOMM20 (translocase of outer mitochondrial membrane 20, ThermoFisher, Waltham, MA, USA) primary antibody, then Alexa Fluor 488 secondary antibody and Hoechst 33,342 (Sigma-Aldrich, St. Louis, MI, USA). They were mounted with Vectashield H-1000 antifade mounting medium (Vector Laboratories, Newark, CA, USA) for mitochondrial analysis by super-resolution microscopy. Super-resolution microscopy using the 3D-structured illumination microscopy (3D-SIM) technique was performed using a DeltaVision OMX V4 Blaze microscope (GE Healthcare, Chicago, IL, USA) with the BGR-FR filter drawer, an Olympus Plan Apochromat 100×/NA 1.40 oil immersion objective lens and liquid-cooled Photometrics Evolve EM-CCD cameras. Fifteen images per z-section per channel were acquired with a z-spacing of 0.125 μm. Structured illumination reconstruction and chromatic alignment were done using softWoRx software (GE Healthcare, Chicago, IL, USA). 3D reconstruction for volume measurement was performed in Imaris (Bitplane, Belfast, UK).

### 2.7. Statistical Analysis

Statistics were analysed using GraphPad Prism. Stimulated T cells were matched within donors according to subset and stimulation time for comparisons. Paired two-way ANOVA tests were used followed by Tukey’s post hoc analysis. The Mann–Whitney test was used to test for significant differences between young and aged T cell subsets and presented in boxplots. Exact *p*-values are identified using * (* *p* < 0.05; ** *p* < 0.01; *** *p* < 0.001).

## 3. Results

### 3.1. Dynamics of Mitochondrial Fission and Fusion Are Unchanged in CD4+ TEMRA Cells Compared to Other CD4+ T Cell Subsets

**Figure 2:**
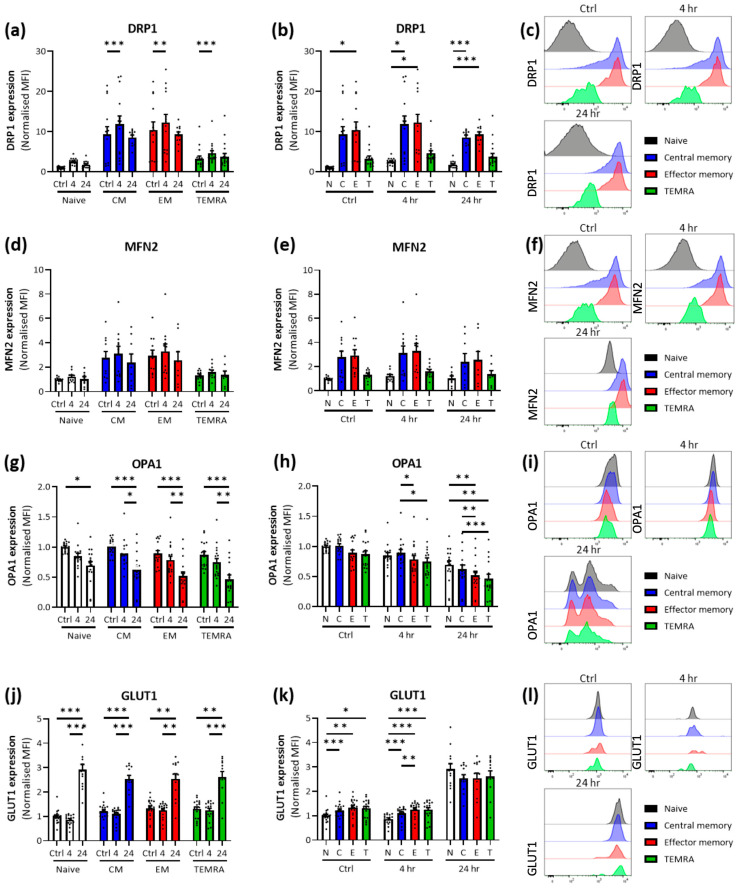
Flow cytometry analysis of proteins controlling mitochondrial dynamics and GLUT1 in CD4+ T cells. (**a**) Change in DRP1 expression with OKT3 stimulation (*n* = 17); (**b**) comparison of DRP1 expression between subsets (*n* = 17); (**c**) histogram of DRP1 expression in a representative sample; (**d**) change in MFN2 expression with OKT3 stimulation (*n* = 17); (**e**) comparison of MFN2 expression between subsets (*n* = 17); (**f**) histogram of MFN2 expression in representative sample; (**g**) change in OPA1 expression with OKT3 stimulation (*n* = 18); (**h**) comparison of OPA1 expression between subsets (*n* = 18); (**i**) histogram of OPA1 expression in a representative sample; (**j**) change in GLUT1 expression with OKT3 stimulation (*n* = 19); (**k**) comparison of GLUT1 expression between subsets (*n* = 19); (**l**) histogram of GLUT1 expression in a representative sample. (Mean+SEM; two-way repeated measures ANOVA with Tukey post hoc analysis; * *p* < 0.05 ** *p* < 0.01 *** *p* < 0.001; expression normalized to unstimulated naïve cells.)

Alterations in mitochondrial dynamics following OKT3 stimulation were measured by expression of DRP1 (dynamin-related protein 1) and MFN2 (mitofusin 2) via flow cytometry (Figure 2a–f). Fluorescence of CD4+ CM (CD27+ CD45RA-), EM (CD27- CD45RA-) and TEMRA (CD27- CD45RA+) were compared to CD4+ naïve T cells (CD27+ CD45RA+) to allow for comparison between each of the groups. Following 4 h of OKT3 stimulation, DRP1 expression was significantly increased, as expected within all three CD4+ memory subsets (Figure 2b,c). CD4+ CM and EM T cells exhibited increased expression of DRP1 following 4 h of OKT3 stimulation compared to naïve cells (Figure 2b,c). This trend was also observed after 24 h of OKT3 stimulation in CD4+ CM and EM T cells. CD4+ EM T cells displayed increased baseline expression of DRP1 prior to OKT3 stimulation compared to naïve T cells (Figure 2b). Significant alterations in MFN2 expression were not observed following OKT3 stimulation over the 4 or 24 h time course, suggesting that fission is the main mitochondrial dynamic during T-cell stimulation (Figure 2d–f). Our analysis of mitochondrial fission and fusion proteins DRP1 and MFN2 shows that CD4+ TEMRA cells behave similarly to CD4+ naïve and CD4+ memory cells in response to stimulation.

### 3.2. Optic Atrophy Protein 1 (OPA1) Expression Is Reduced in CD4+ EM and TEMRA Cells following OKT3 Stimulation

Cristae remodelling plays a significant role in T cell stimulation and has previously been shown to be differentially regulated between T cell subsets within the CD4+ and CD8+ compartments. Therefore, the expression of cristae remodelling optic protein atrophy 1 (OPA1) was analysed by flow cytometry within naïve and memory CD4+ T cells. Following 24 h of OKT3 stimulation, OPA1 expression was significantly decreased in all CD4+ subsets, including naïve cells (Figure 2g).

No differences in OPA1 expression were observed between the four CD4+ T cell subsets before OKT3 stimulation (Figure 2h). After 4 h of OKT3 stimulation, OPA1 was significantly decreased in CD4+ EM and TEMRA cells compared to the CM cells (Figure 2h). This finding also extended to the 24 h time point following OKT3 stimulation, where CD4+ EM and TEMRA showed decreased OPA1 expression compared to both CD4+ naïve and CM T cells (Figure 2h). Interestingly, OKT3 stimulation for 24 h resulted in the appearance of two peaks of OPA1 expression (Figure 2i). In summary, we showed that CD4+ TEMRA cells have significantly lower levels of OPA1 than other CD4+ T cell subtypes.

### 3.3. Glucose Transporter 1 (GLUT1) Expression Is Increased in CD4+ CM, EM and TEMRA Cells during the Early Stages of OKT3 Stimulation

Glycolysis plays a significant role in engaging effector function following stimulation; therefore, we measured the extracellular expression of GLUT1 by flow cytometry. No significant upregulation of GLUT1 expression on the cell surface was observed within the first 4 h of OKT3 stimulation (Figure 2j,l). As for CD4+ naïve T cells, CD4+ CM, CD4+ EM and CD4+ TEMRA all upregulated GLUT1 expression on the cell surface following 24 h of OKT3 stimulation (Figure 2k,l). Prior to stimulation, memory CD4+ T cells displayed increased surface GLUT1 expression compared to CD4+ naïve T cells; this includes CM, EM and TEMRA subsets (Figure 2k,l).

The increased expression of GLUT1 was also observed following 4 h of OKT3 stimulation in CD4+ CM, EM and TEMRA compared to naïve cells. Interestingly, GLUT1 upregulation in CD4+ memory T cell subsets was diminished after 24 h OKT3 stimulation, suggesting that the increase in GLUT1 expression is transient (Figure 2k). Considering these findings, we show that while GLUT1 expression is induced in response to stimulation all in CD4+ subsets, CD4+ TEMRA and other memory cells have increased basal levels of GLUT1.

### 3.4. Mitochondrial Mass Is Retained within the CD4+ TEMRA Cells, While Mitochondrial Membrane Potential Is Reduced

**Figure 3:**
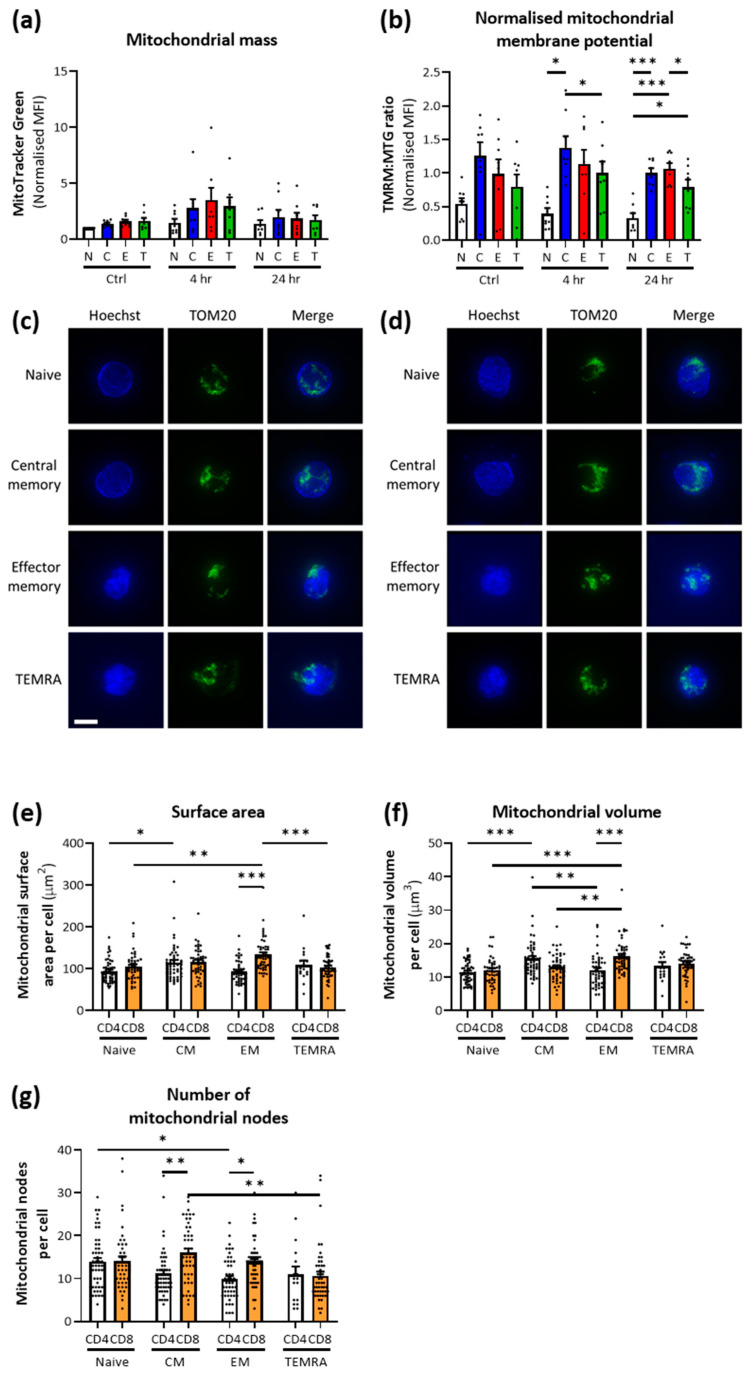
Analysis of mitochondrial mass and membrane potential in CD4+ and CD8+ T cells. Flow cytometry analysis of (**a**) comparison of MitoTracker Green staining between CD4+ T cell subsets (*n* = 8; expression normalised to unstimulated naïve cells) and (**b**) comparison of mitochondrial membrane potential between CD4+ T cell subsets (*n* = 8; TMRM staining normalised to mitochondrial mass). Representative super-resolution microscopy imaging of sorted (**c**) CD4+ T cells and (**d**) CD8+ T cells (scale bar = 10 μm; middle plane image), and analysis of (**e**) mitochondrial surface area (*n* = 40), (**f**) mitochondrial volume (*n* = 40) and (**g**) the number of mitochondrial nodes (*n* = 40). (Mean + SEM; two-way repeated measures ANOVA with Tukey post hoc analysis; * *p* < 0.05 ** *p* < 0.01 *** *p* < 0.001.)

MitoTracker Green (MTG) and tetramethylrhodamine methyl ester (TMRM) were utilised to measure mitochondrial mass and membrane potential in T cells following stimulation. No changes were observed in mitochondrial mass following OKT3 stimulation of 4 and 24 h (Figure 3a). Despite previous observations in other studies, no differences in mitochondrial mass were observed between CD4+ naïve and memory CD4+ T cells.

However, differences in mitochondrial membrane potential were noted. Mitochondria in CD4+ CM cells displayed significantly higher membrane potential than naïve cells after 4 and 24 h of OKT3 stimulation. However, no significant differences were observed before stimulation (Figure 3b). Following 24 h of OKT3 stimulation, this trend was also observed in all CD4+ T cell subsets.

While memory cells were observed to increase mitochondrial membrane potential following OKT3 stimulation, TEMRA cells did not increase their membrane potential to the same extent as CM and EM cells (Figure 3b). Mitochondrial membrane potential was reduced in CD4+ TEMRA cells compared to CD4+ CM following 4 h of OKT3 stimulation. Similarly, mitochondrial membrane potential was reduced in CD4+ TEMRA compared to CD4+ EM cells following 24 h of OKT3 stimulation. These data suggest that CD4+ TEMRA cells regulate their mitochondrial membrane potential differently from CD4+ CM and EM. Taken together, our findings show that mitochondrial membrane potential is increased in CD4+ memory cells compared to naïve cells. However, it is reduced in CD4+ TEMRA compared to central and effector memory subtypes.

### 3.5. Analysis of CD8+ and CD4+ T Cell Subsets Reveals Differences in Mitochondrial Structure

To visualise mitochondria within CD4+ and CD8+ T cells, naïve, CM, EM and TEMRA cells were sorted by FACS and imaged using super-resolution microscopy. Cells were stained with TOMM20 and Hoescht to distinguish the mitochondria and nucleus of each cell. Representative images of CD4+ and CD8+ cells are shown in Figure 3c,d, respectively. The 3D reconstruction revealed that mitochondria in CD4+ CM cells had increased mitochondrial volume and surface area compared to naïve T cells (Figure 3e,f). However, this finding was not observed for CD8+ CM and naïve T cells. Additionally, CD4+ EM cells showed decreased mitochondrial volume compared to CD4+ CM cells (Figure 3f).

The surface area and volume of the mitochondria were significantly reduced in CD4+ EM compared to CD8+ EM. However, there were no changes in mitochondrial content regarding the surface area and volume between naïve T cells, and CM and TEMRA within the CD4+ and CD8+ compartments (Figure 3e,f). When calculating the number of mitochondria by calculating the number of distinct nodes, a reduction in the mitochondrial number was noted for CD4+ CM and EM cells but not CD8+ CM and EM cells (Figure 3g). CD4+ EM showed a reduced number of mitochondrial nodes compared to CD4+ naïve cells. Taken together, our imaging analysis shows that CD4+ and CD8+ TEMRA have a mitochondrial landscape similar to that of naïve T cells, with memory cells often showing alterations in mitochondrial volume, surface area and number in both the CD4+ and CD8+ compartments.

### 3.6. Age-Associated Differences in Mitochondrial Mass Are Unique to the CD4 TEMRA Subset

**Figure 4:**
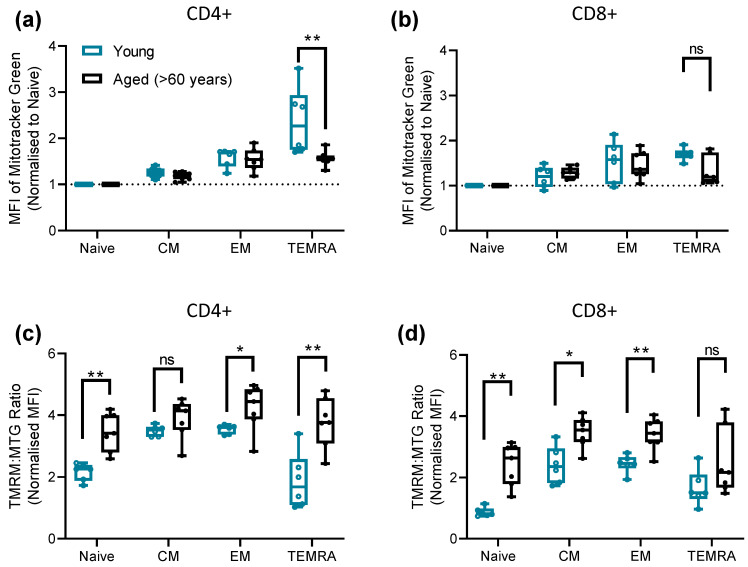
Age-associated differences in mitochondrial mass are unique to the CD4 TEMRA subset. Flow cytometry analysis was used to compare MitoTracker Green staining between (**a**) CD4+ T cell subsets and (**b**) CD8+ T cell subsets. Flow cytometry analysis was used to compare mitochondrial membrane potential between (**c**) CD4+ T cell subsets and (**d**) CD8+ T cell subsets. (**a**–**d**) Mann–Whitney test was used to test for significant differences between young (*n* = 6; average age = 30.1 years) and aged (*n* = 7; average age = 71.2 years) for each T cell subset. Data presented in boxplots; ns = non-significant, * *p* < 0.05 ** *p* < 0.01).

Considering that ageing and senescence play a significant role in T cell biology, we next looked into the mitochondrial mass and membrane potential of various CD4 and CD8 T cell subsets from the peripheral blood of young and aged individuals. Based on our flow cytometric analysis, CD4 TEMRA had a more significant mitochondria mass in young individuals than in the elderly (Figure 4a), which was not as significantly observed for CD8 TEMRA (Figure 4b). Regarding mitochondrial potential, there was a significant increase in mitochondrial membrane potential across various CD4 and CD8 T cell subsets of aged individuals compared to young individuals, except for CD4 CM and CD8 TEMRA cells (Figure 4c,d). Taken together, our data show age-associated differences in mitochondria that would influence the general metabolism of the T cell subsets.

## 4. Discussion

We report, alongside others, that TEMRA cells exhibit altered mitochondrial control [4,13,16,22]. Previous research has suggested through clustering analysis that two populations of TEMRA cells exist within the CD8+ T cell population, one with a close association with naïve cells and the other with a close association with EM cells [8]. This phenomenon is also somewhat true of the CD4+ TEMRA population, whereby CD4+ TEMRA show similarities to the naïve and memory cell populations regarding mitochondrial characteristics.

Studies of MFN2 and DRP1 in T cells are limited, let alone in rare populations such as CD4+ TEMRA cells. Here, we present data on mitochondrial fission and fusion within human CD4+ T cells. The expression levels of these proteins have previously been compared in CD8+ T cells derived from C57BL/6 mice, where increased expression of Mfn2 and decreased phosphorylation of Drp1 was observed in memory T cells compared to naïve T cells [23]. However, in this study, the opposite trend was observed, with human memory CD4+ T cells showing increased expression of DRP1 and no significant alterations in MFN2 levels in humans. Whether these alterations are species- or CD4/8-specific is unknown, but warrants further research.

Interestingly, unlike CM and EM CD4+ memory T cells, DRP1 expression was not upregulated in CD4+ TEMRA cells compared to in naïve cells. As DRP1 has been shown to support metabolic reprogramming within effector cells following activation, our findings suggest that CM and EM CD4+ T cells may be primed to respond more quickly to stimulation [24]. TEMRA cells show no significant upregulation of DRP1 compared to either naïve cells or CM and EM cells, suggesting that these cells may also, to a lesser extent, be primed for secondary stimulation following infection.

Memory development has previously been shown to be dependent on OPA1, which controls the fusion of the inner mitochondrial membrane, as T cells lacking this protein show deformed and disorganised cristae within the mitochondria [23]. Our study showed a decrease in OPA1 in CD4+ naïve, CM, EM and CD4+ TEMRA cells following OKT3 stimulation. This was followed in parallel by GLUT1 upregulation. The change in the OPA1:GLUT1 ratio implies the engagement of glycolysis following OKT3 stimulation, which simultaneously disengaged oxidative phosphorylation mediated by OPA1. This finding reflects the changing metabolic requirements of effector T cells following stimulation observed elsewhere [4,13,25]. CD4+ T cells express four of the thirteen GLUT family members (GLUT1, 3, 6 and 8), with GLUT1 being the most highly expressed following activation [26]. Interestingly, due to the importance of GLUT1 in maintaining CD4+ T cell metabolism and function, CD4+ TEMRA cells showed no defect in GLUT1 levels and expressed similar levels to those expressed in CD4+ EM T cells. As previously reported, memory CD4+ T cells expressed higher levels of GLUT1 at baseline and during the early stage (4 h) of OKT3 stimulation [22,26,27]. On the other hand, CD8+ TEMRA showed reduced expression of GLUT1 [4]. Interestingly, OPA1 expression was downregulated within EM and TEMRA cells compared to naïve and CM following OKT3 stimulation. GLUT1 expression was shown to be increased in CD4+ T cells after OKT3 stimulation, supporting the offset of oxidative phosphorylation and glycolysis within these cells.

Our findings suggest that mitochondrial regulation differs between the CD8+ and CD4+ T cell compartments. While CD8+ TEMRA cells have reduced mitochondrial mass compared to EM T cells, this is not true for the CD4+ T cell subset. We did, however, report a reduction in mitochondrial membrane potential in CD4+ TEMRA, which has also been observed for CD8+ TEMRA [4]. This finding was demonstrated following OKT3 stimulation, but not at baseline, and may affect the ability of CD4+ TEMRA to produce IL-21 and IL-4 and reduce effector function [28]. Decreased production of IL-21 and IL-4 has been reported with human ageing, impairing memory CD4+ T cell responses to infection and vaccination efficacy [29]. We observed a significant reduction in the mitochondrial surface area between CD8+ EM and TEMRA cells using super-resolution microscopy, supporting previous findings of CD8+ T cell subset mitochondrial analysis [4]. This reduction was not observed within the CD4+ T cell compartment, supporting our flow cytometric analysis of mitochondrial mass.

Most studies to date have focused on comparing CD4+ TEMRA with CD8+ TEMRA, even though the roles of CD4+ and CD8+ T cells are fundamentally different. CD4+ TEMRA cells have almost double the mitochondrial content of CD8+ TEMRA, and are slower to develop a senescent phenotype in comparison [16]. They have fewer mitochondrial nodes than CD8+ TEMRA, and higher expression of PGC1a, suggesting that they are more amenable to altering their energy requirements [16]. However, CD4+ TEMRA also have a higher proportion of hyperpolarised mitochondria linked to increased ROS production and DNA damage [16]. Few studies have looked into CD4+ TEMRA compared to other CD4+ T cell subsets and what effect this may have on their phenotype.

Reductions in mitochondrial mass and the regulation of mitochondrial biogenesis have been reported in T cells with ageing [30,31,32]. From our comparison of young and aged individuals, a reduction in mitochondrial mass was mainly observed in CD4+ TEMRA. Across several subsets of CD4 and CD8 T cells, mitochondrial membrane potential was elevated at baseline, further suggesting hyperpolarisation of the mitochondria to cope with age-driven metabolic changes. On the other hand, we showed that CD4+ TEMRA do not increase their mitochondrial membrane potential as drastically as CM and EM cells upon anti-CD3 stimulation, which suggests that their mitochondrial capacity is impaired and may affect responses to infection in the elderly.

## 5. Conclusions

**Figure 5:**
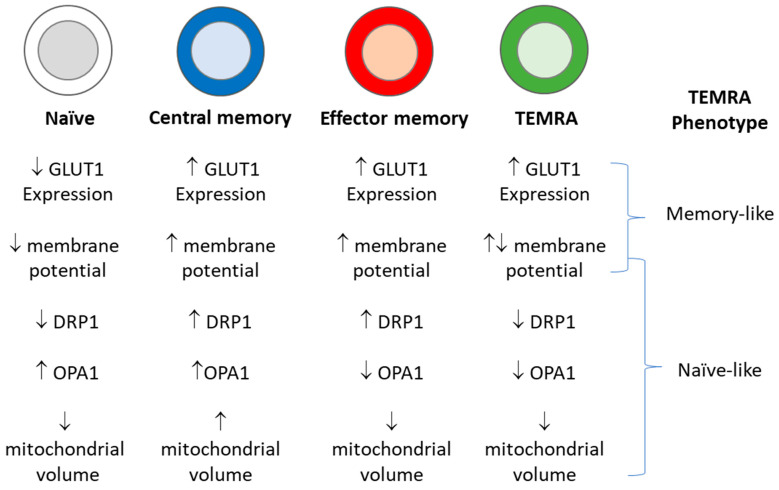
Summary of mitochondrial findings in CD4+ naïve and memory T cells, including terminal effector memory T cells re-expressing CD45RA (TEMRA) cells. Arrows indicate an increase or decrease in the respective parameter.

In conclusion, we showed that CD4+ TEMRA exhibits altered mitochondrial dynamics compared to both CD4+ naïve cells and CD4+ CM and EM cells. Interestingly, TEMRA cells could be both naïve-like and memory-like in terms of their mitochondrial phenotype (Figure 5), which at the same time could be influenced by ageing. Our present study provides insights into the metabolism of TEMRA cells, which could be of considerable importance, particularly in the study of age-related disorders. Future studies should also continue to uncover how mitochondrial dynamics influences T cell function, particularly in response to infection and influenza vaccination and the SASP phenotype.

## Data Availability

Data supporting the reported results have been deposited via PURE into ePrints Soton (https://eprints.soton.ac.uk/, accessed on 10 December 2022).
